# Supplementary material for: Recent Advances in the Behavioral Ecology of European Plethodontid Salamanders
Source: Animals (Basel). 2023 Nov 27;13(23):3667. doi: 10.3390/ani13233667 (PMC10705341; doi:10.3390/ani13233667)
Supplement: Supplementary file 1 [file animals-13-03667-s001.zip › animals-2682639-supplementary.pdf]

Supplementary TableS1 **Complete list of *Speleomantes* paper 2006-2022**
papers highlieted in yellow were analised

Year	Authors	Title	Reference	Behavioral Ecology topic	Type of study
2006	Vignoli L., Caldera F., Bologna M.A	Trophic niche of cave populations of Speleomantes	Journal Natural History, 40: 29-	Foraging	observational
2006	De Martini L., Oneto F., Pastorino M.V., Salvidio S., Buriola E., Bona F	A non-lethal method to sample gastrointestinal parasites from terrestrial salamanders	Amphibia-Reptilia, 27: 278-280		
2006	Salvidio S., Bruce R.C.	Sexual dimorphism in two species of European plethodontid salamanders, genus Speleomantes	Herpetological Journal, 16: 9-14		
2006	Oneto F., Pastorino M.V., Salvidio S., De Martini L.	Studio delle parassitosi intestinali in Speleomantes strinaii con metodiche non traumatizzantiStudio delle parassitosi intestinali in Speleomantes strinaii con	Atti V Congresso Nazionale Societas Herpetologica Italica, Calci, 29 settembre - 3 ottobre		
2007	Salvidio S.	Population dynamics and regulation in the cave salamander Speleomantes strinatii	Naturwissenschaften, 94: 396-400		
2007	Pastorelli C., Laghi P.	Predation on Speleomantes italicus (amphibia: Caudata: Plethodontidae) by Meta menardi (Arachnida: Araneae: Metidae)	Atti VI Congresso Nazionale Societas Herpetologica Italica, Roma, 27 settembre - 1 ottobre 2006. pp. 45-48. Bologna M.A.,	Interactions with predators	observational
2007	Crochet P.-A.	Nomenclature of European plethodontid salamanders: Speleomantes Dubois, 1984 has precedence over	Amphibia-Reptilia, 28: 170-172		
2008	Carranza S., Romano A., Arnold E.N., Sotgiu G.	Biogeography and evolution of European cave salamanders, Hydromantes (Urodela: Plethodontidae), inferred from mtDNA sequences	Journal of Biogeography, 75: 724-738		
2008	Salvidio S.	Temporal variation in adult sex ratio in a population of the terrestrial salamander Speleomantes strinatii	Herpetological Journal, 18: 66-68		
2008	Vignoli L., Caldera F., Bologna M.A.	Spatial niche of the Italian cave salamander, Speleomantes italicus (Dunn, 1923) (Plethodontidae, Amphibia), in a subterranean system of Central Italy	Italian Journal of Zoology, 55: 69-75	Intraspecific social behavior, courtship, mating and	observational
2008	van der Meijden A., Chiary Y., Mucedda M., Carranza S., Corti C., Veith M.	Phylogeny of Sardinian Cave salamanders	Herpetologia Sardiniae. Pp. 367-369. Corti C. (ED.). Edizioni Belvedere, Latina (Italy).		

2008	Tessa G., Sotgiu G., Repetto R., Giacoma C., Cazzaniga E., Favelli M., Doglio S., Candiotto A., Bovero S.	Longevity and population dynamics in Speleomantes imperialis sarrabusensis (Southern Sardinia Italy)	Herpetologia Sardiniae. Pp. 475-478. Corti C. (ED.). Edizioni Belvedere, Latina (Italy).		
2009	van der Meijden A., Chiari Y., Mucedda M., Carranza S., Corti C., Veith M.	Phylogenetic relationships of Sardinian cave salamanders, genus Hydromantes, based on mitochondrial and nuclear DNA sequence data	Molecular Phylogenetics and Evolution, 51: 399-404		
2010	Romano A., Amat F., Rivera X., Sotgiu G., Carranza S.	Evidence of tail autotomy in the European plethodontid Hydromantes (Atylodes) genei (Temmick and Schlegel, 1838) (Amphibia: Urodela: Plethodontidae)	Acta Herpetologica, 5: 199-205	Interactions with predators	observational
2010	Oneto F., Ottonello D., Pastorino M.V., Salvidio S.	Posthatching parental care in salamanders revealed by infrared video surveillance	Journal of Herpetology, 44: 649-653	Intraspecific social behavior, courtship, mating and	observational
2010	Adams D.C., Nistri A.	Ontogenetic convergence and evolution of foot morphology in European cave salamanders (Family: Plethodontidae)	BMC Evolutionary Biology, 10: 216		
2010	Lindström J., Reeve R., Salvidio S.	Bayesian salamanders: Analysing the demography of an underground population of the European plethodontid Speleomantes strinatii with state-space models	BMC Ecology, 10: 4		
2010	Sguanci Corti C., Berti R., Vanni S., Salvidio S.	Difesa del territorio in Speleomantes italicus (Dunn, 1923).	Atti VIII Congresso Nazionale Societas Herpetologica Italica. Pp. 253-258. Di Tizio L., Di Cerbo A.R., Di Francesco N., Cameli A. (Eds)	Intraspecific social behavior, courtship, mating and parental cares	experimental
2012	Salvidio S., Romano A., Fabrizio O., Dario O., Roberta Michelon	Different season, different strategies: Feeding ecology of two syntopic forest-dwelling salamanders	Acta Oecologica, 43: 42-50	Foraging tactics	observational
2012	Ficetola G.F., Pennati R., Manenti R.	Do cave salamanders occur randomly in cavities? An analysis with Hydromantes strinatii	Amphibia Reptilia, 33: 251-259		
2012	Crovetto F., Romano A., Salvidio S.	Comparison of two non-lethal methods for dietary studies in terrestrial salamanders	Wildlife Research, 39: 266-270		

2012	Chiari Y., van der Meijden A., Mucedda M., Lourenço J.M., Hochkirch A., Veith M.	Phylogeography of Sardinian cave salamanders (genus Hydromantes) is mainly determined by geomorphology	PLoS ONE, 7: e32332		
2012	Renet J., Tordjman P., Gerriet O., Madelaine E.	Le Spélerpès de Strinati, Speleomantes strinatii (Aellen, 1958) (Amphibia, Urodela, Plethodontidae) : répartition des populations autochtones en France et en Principauté de Monaco	Bulletin Societé Herpétologique de France, 141: 3-22		
2013	Wagner N., Chiari Y., Mucedda M., Van Der Meijden A., Veith M.	No detection of the pathogen Batrachochytrium dendrobatidis in sardinian cave salamanders, genus Hydromantes	Amphibia Reptilia, 34: 136-141		
2013	Pasmans F., Van Rooij P., Blooi M., Tessa G., Bogaerts S., Sotgiu G., Garner T.W.J., Fisher M.C., Schmidt B.R., Woeltjes T., Beukema W., Bovero S., Adriaensen C., Oneto F., Ottonello D., Martel A., Salvidio S.	Resistance to Chytridiomycosis in European Plethodontid Salamanders of the Genus Speleomantes	PLoS ONE, 8: e63639		
2013	Salvidio S.	Homing behaviour in Speleomantes strinatii (Amphibia Plethodontidae): A preliminary displacement experiment	North-Western Journal of Zoology, 9: 429-432	Intraspecific territoriality and homing	experimental
2013	Ficetola G.F., Pennati R., Manenti R.	Spatial segregation among age classes in cave salamanders: Habitat selection or social interactions?	Population Ecology, 55: 217-226	Intraspecific social behavior, courtship, mating and	observational
2013	Oneto F., Ottonello D., Pastorino M.V., Salvidio S.	Nuovi dati sul comportamento di cura parentale nei Caudati: Hydromantes (Speleomantes) strinatii (Amphibia, Plethodontidae).	Atti IX Congresso Nazionale della Societas Herpetologica Italica, (Bari - Conversano, 26-30 settembre 2012). Pp. 204-207. Scillitani G., Liuzzi C., Lorusso L.,	Intraspecific social behavior, courtship, mating and	experimental

2013	Ottonello D., Oneto F.	Contributo allo studio della popolazione di Hydromantes (Speleomantes) strinatii delle Grotte del Bandito (Alpi Marittime, Italia nordoccidentale)	Atti IX Congresso Nazionale della Societas Herpetologica Italica. Bari - Conversano, 26-30 settembre 2012. Pp. 241-244. Scillitani G., Liuzzi C., Lorusso L., Mastropasqua F., Ventrella P. (Eds)		
2014	Salvidio S., Oneto F., Ottonello D., Costa A., Romano A.	Trophic specialization at the individual level in a terrestrial generalist salamander	Canadian Journal of Zoology, 93: 79-83	Foraging tactics	observational
2014	Wake D.B.	The enigmatic history of the European, Asian and American plethodontid salamanders	Amphibia Reptilia, 34: 323-336		
2014	Cimmaruta R., Forti G., Lucente D., Nascetti G.	Thirty years of artificial syntopy between Hydromantes italicus and H. ambrosii ambrosii (Amphibia, Plethodontidae)	Amphibia Reptilia, 34: 413-420		
2014	Lunghi E., Manenti R., Manca S., Mulargia M., Pennati R., Ficetola G.F.	Nesting of cave salamanders (Hydromantes flavus and H. italicus) in natural environments	Salamandra, 50: 105-109	Intraspecific social behavior, courtship, mating and	observational
2014	Manenti R.	Dry stone walls favour biodiversity: A case-study from the Appennines	Biodiversity and Conservation, 23: 1879-1893		
2014	Lunghi E., Manenti R., Ficetola G.F.	Do cave features affect underground habitat exploitation by non-troglobite species?	Acta Oecologica, 55: 29-35		
2014	Oneto F., Ottonello D., Pastorino M.V. Salvidio S.	Maternal care and defence of young by the plethodontid salamandre Speleomantes strinatii (Aellen, 1951)	Scripta Herpetologica. Studies on Amphibians and Reptiles in honour of Benedetto Lanza. Pp. 129- [illegible]	Intraspecific social behavior, courtship, mating and	experimental
2015	Salvidio S., Crovetto F., Adams D.C.	Potential rapid evolution of foot morphology in Italian plethodontid salamanders (Hydromantes strinatii) following the colonization of an artificial cave	Journal of Evolutionary Biology, 28: 1403_1409		
2015	Cimmaruta R., Lucente D., Nascetti G	Persistence, isolation and diversification of a naturally fragmented species in local refugia: The case of hydromantes strinatii	PLoS ONE, 10: e0131298		

2015	Lunghi E., Murgia R., De Falco G., Buschettu S., Mulas C., Mulargia M., Canedoli C., Manenti R., Ficetola G.F	First data on nesting ecology and behaviour in the imperial cave salamander Hydromantes imperialis	North-Western Journal of Zoology, 11: 324-330	Intraspecific social behavior, courtship, mating and	observational
2015	Lunghi E., Manenti R., Ficetola G.F.	Seasonal variation in microhabitat of salamanders: Environmental variation or shift of habitat selection?	PeerJ, 8: 1122		
2015	Costa A., Crovetto F., Salvidio S.	Assessing salamander abundance and density with N-mixture models: preliminary results on a Speleomantes strinatii (Aellen, 1858) population	Atti X Congresso Nazionale Societas Herpetologica Italica. Genova, 15-18 ottobre 2014. Pp. 63-69. Doria G., Poggi R. Salvidio S. ravano M. (Eds).		
2015	Ficetola G.F., Lunghi E., Pennati R., Manenti R.	Variation of ecological niche in cave salamanders (genus Hydromantes)	Atti X Congresso Nazionale Societas Herpetologica Italica. Genova, 15-18 ottobre 2014. Pp. 71-76. Doria G., Poggi R. Salvidio S. ravano M. (Eds).		
2015	Lunghi E., Manenti R., Ficetola G.F.	Distribuzione e fenologia del geotrtitone (Hydromantes italicus) nell'Appennino centro-settentrionale: dati preliminari sulla presenza e sull'osservabilità in ambiente epigeo	Atti X Congresso Nazionale Societas Herpetologica Italica. Genova, 15-18 ottobre 2014. Pp. 91-98. Doria G., Poggi R. Salvidio S. ravano M. (Eds).		
2015	Salvidio S., Oneto F., Ottonello D., Pastorino M.V.	Monitoraggio a lungo termine del geotritone *Speleomantes strinatii* nella Stazione Biospeleologica di San Bartolomeo di Besolagno (GE).	Atti 22° Congresso Nazionale di Speleologia – Euro Speleo Forum 2015, 30 maggio – 2 giugno 2015, Pertosa – Auletta (SA). Memorie dell'Istituto Italiano di Speleologia. Pp. 423-428. De Nitto L., Maurano F., Parise M. (Eds)		
2015	Rivera X., Amat F., Sotgiu G., Romano A.	Patrons de coloració de la salamandra Atylodes genei (Temminck & Schlegel, 1838) (Caudata, Plethodontidae	Butlletì dela Societat Catalana d'Herpetologia 22: 77-93		

2016	Lucente D., Renet J., Gailledrat M., Tillet J., Nascetti G., Cimmaruta R.	A new population of European cave salamanders (genus Hydromantes) from west-central France: Relict or introduction?	Herpetological Bulletin, 138: 21-23		
2016	Costa A., Crovetto F., Salvidio S.	European plethodontid salamanders on the forest floor: Local abundance is related to fine-scale environmental factors	Herpetological Conservation and Biology, 11: 344-349		
2016	Lunghi E., Manenti R., Canciani G., Scarì G., Pennati R., Ficetola G.F.	Thermal equilibrium and temperature differences among body regions in European plethodontid salamanders	Journal of Thermal Biology, 60: 79-85		
2016	Lunghi E., Mulargia M., Mulargia M.	Evidence of malformation in the European cave salamander, Hydromantes flavus	Herpetological Bulletin, 135: 34-35		
2016	Bruni G., Novaga r., Fiacchini D., Spilinga C., Domeneghetti D.	Updated distribution of Hydromantes italicus Dunn, 1923 (Caudata Plethodontidae): a review with new [illegible]	Biodiversity journal, 7: 347-352		
2016	Manenti, R., Lunghi, E., Canedoli, C., Bonaccorsi, M., Ficetola, G.F.	Parasitism of the leech, Batracobdella algira (MOQUIN-TANDON, 1846), on Sardinian cave salamanders (Genus Hydromantes) (Caudata: Plethodontidae)	Herpetozoa, 29: 27-35	Interactions with predators and parasites	observational
2017	Lunghi E., Ficetola G.F., Barzaghi B., Vitillo C., Mulargia M., Manenti R.	Melanism in European plethodontid salamanders (Amphibia, plethodontidae, hydromantes) the first report for Latium (Italy)	Spixiana, 40: 157-160		
2017	Salvidio S., Palumbi G., Romano A., Costa A.	Safe caves and dangerous forests? Predation risk may contribute to salamander colonization of subterranean habitats	Science of Nature, 104: 20	Interactions with predators and parasites	experimental
2017	Lunghi E., Monti A., Binda A., Piazzi I., Salvadori M., Cogoni R., Riefolo L.A., Biancardi C., Mezzadri S., Avitabile D., Ficetola G.F., Mulargia M., Manca S., Blaimont P., Di Cerbo A.R., Manenti R.,	Cases of albinism and leucism in amphibians in Italy: New reports	Natural History Sciences, 4: 73-80		
2017	Lunghi E., Veith M.	Are visual implant alpha tags adequate for individually marking European cave salamanders (genus Hydromantes)?	Salamandra, 53: 541-544		
2017	Adams D.C., Korneisel D., Young M., Nistri A.	Natural history constrains the macroevolution of foot morphology in european plethodontid salamanders	American Naturalist, 190: 292-297		

2017	Salvidio S., Pasmans F., Bogaerts S., Martel A., Van De Loo M., Romano A.,	Consistency in trophic strategies between populations of the Sardinian endemic salamander Speleomantes imperialis	Animal Biology, 67: 1-16	Foraging tactics	observational
2017	Lunghi E., Ceccolini F., Cianferoni F., Cornago L., Mulargia M.,Cogoni R., Manenti R., Corti C., Ficetola G.F.	Dati preliminari sulla dieta autunnale dei geotritoni della Sardegna (genere Speleomantes)	Atti XI Congresso Nazionale Societas Herpetologica Italica. Trento, 22-25 settembre 2016. Pp. 177-182. Menegon M., Rodriguez-Prieto A., Deflorian Maria Chiara (eds).	Foraging tactics	observational
2017	Cameli SA., Ferri V., De Luca L., Pandolfi M., Di Toro F., Soccini C.	Speleomantes italicus (Dunn, 1923) in Abruzzo: nuove segnalazioni e proposte di conservazione	Atti XI Congresso Nazionale Societas Herpetologica Italica. Tento, 22-25 settembre 2016. Pp. 29-34. Menegon M., Rodriguez-Prieto A., Deflorian Maria Chiara (eds).		
2017	Muraro M., Manenti R., Pennati R., Lu	Primi dati di uno studio di popolazione del geotrtitone italiano Speleomantes italicus (Dunn, 1923) in ambiente ipogeo: valutazione dell'affidabilità del removal sampling	Atti XI Congresso Nazionale Societas Herpetologica Italica. Tento, 22-25 settembre 2016. Pp. 141-146. Menegon M., Rodriguez-Prieto A., Deflorian Maria Chiara (eds).		
2017	Oneto F., Salvidio S., Ottonello D., Pastorino M.V.	Comportamento difensivo e aggressività nel geotritone di Strinati Speleomantes strinatii (Aellen, 1958).	Atti XI Congresso Nazionale Societas Herpetologica Italica. Tento, 22-25 settembre 2016. Pp. 183-189. Menegon M., Rodriguez-Prieto A., Deflorian	Intraspecific social behavior, courtship, mating and	experimental
2017	Buriola E., Oneto F., Ottonello D., Pastorino M.V., Salvidio S.	Nuovi dati su Cestodi parassiti dei Pletodontidi europei: contributo per una revisione zoogeografica e sistematica	Atti XI Congresso Nazionale Societas Herpetologica Italica. Tento, 22-25 settembre 2016. Pp. 391-397. Menegon M., Rodriguez-Prieto A., Deflorian Maria Chiara (eds).		
2018	Ficetola G.F., Lunghi E., Canedoli C., Padoa-Schioppa E., Pennati R., Manenti R.	Differences between microhabitat and broad-scale patterns of niche evolution in terrestrial salamanders	Scientific Reports, 8: 10575		

2018	Ficetola G.F., Barzaghi B., Melotto A., Muraro M., Lunghi E., Canedoli C., Lo Parrino E., Nanni V., Silvia-Rocha I., Urso A., Carretero MA., Salvi D.	N-mixture models reliably estimate the abundance of small vertebrates	Scientific Reports, 8: 10357		
2018	Lunghi E., Manenti R., Mulargia M., Veith M., Corti C., Ficetola G.F.	Environmental suitability models predict population density, performance and body condition for microendemic salamanders	Scientific Reports, 8: 7527		
2018	Lunghi E., Bruni G.	Long-term reliability of visual implant elastomers in the Italian cave salamander (Hydromantes italicus)	Salamandra, 54: 283-286		
2018	Lunghi E., Cianferoni F., Ceccolini F., Veith M., Manenti R., Mancinelli G., Corti C., Ficetola G.F.Lunghi E., Cianferoni F., Ceccolini F., Veith M., Manenti R., Mancinelli G., Corti C., Ficetola G.F.	What shapes the trophic niche of European plethodontid salamanders?	PLoS ONE, 13: e0205672	Foraging tactics	observational
2018	Lunghi E., Cianferoni F., Ceccolini F., Mulargia M., Cogoni R., Barzaghi B., Cornago L., Avitabile D., Veith M., Manenti R., Ficetola G.F., Corti C.	Data Descriptor: Field-recorded data on the diet of six species of European Hydromantes cave salamanders	Scientific Data, 5: 180083		
2018	Lunghi E., Ficetola G.F., Mulargia M., Cogoni R., Veith M., Corti C., Manenti R.	Batracobdella leeches, environmental features and Hydromantes salamanders	International Journal for Parasitology: Parasites and Wildlife, 7: 48-53		
2018	Lunghi E., Corti C., Manenti R., Barzaghi B., Buschettu S., Canedoli C., Cogoni R., De Falco G., Fais F., Manca A., Mirimin V., Mulargia M., Mulas C., Muraro M., Murgia R., Veith M., Ficetola G.F.	Comparative reproductive biology of european cave salamanders (Genus hydromantes): Nesting selection and multiple annual breeding	Salamandra, 54: 101-108	Intraspecific social behavior, courtship, mating and parental cares	observational
2018	Lunghi E., Guillaume O., Blaimont P., Manenti R.	The first ecological study on the oldest allochthonous population of European cave salamanders (Hydromantes sp.)	Amphibia Reptilia, 39: 113-119	Foraging	
2018	Lunghi E., Mascia C., Mulargia M., Corti C.	Is the Sardinian grass snake (Natrix natrix an active hunter in underground environments?	Spixiana, 41: 160.		
2018	Lunghi E.	Ecology and life history of Meta bourneti (Araneae: Tetragnathidae) from Monte Albo (Sardinia, Italy)	PeerJ 6(1):e6049		

2018	Lunghi, E., G. F. Ficetola, M. Mulargia, R. Cogoni, M. Veith, C. Corti and R. Manenti	Batracobdella leeches, environmental features and Hydromantes salamanders.	International Journal for Parasitology: Parasites and Wildlife 7: 48-53.	Interactions with predators and parasites	
2019	Ficetola G.F., Lunghi E., Cimmaruta R., Manenti R.,Ficetola G.F., Lunghi E., Cimmaruta R., Manenti R.,	Transgressive niche across a salamander hybrid zone revealed by microhabitat analyses	Journal of Biogeography, 46: 1342-1354		
2019	Amat F., Rivera X., Romano A., Sotgiu G.	Sexual dimorphism in the endemic Sardinian cave salamander (Atylodes genei)	Folia Zoologica, 68: 61-65		
2019	Lunghi E., Romeo D., Mulargia M., Cogoni R., Manenti R., Corti C., Ficetola G.F., Veith M.	On the stability of the dorsal pattern of European cave Salamanders (genus Hydromantes)	Herpetozoa, 32: 249-253		
2019	Lunghi E., Corti C., Mananti R., [illegible]	Consider species specialism when publishing datasets	Nature Ecology and Evolution, 3: [illegible]		
2019	Renet J., Lepetre L., Champagnon J., Lambret P.	Monitoring amphibian species with complex chromatophore patterns: a non-invasive approach with an evaluation of software effectiveness and reliability	Herpetological Journal, 29: 13-22		
2020	Lunghi E., Giachello S., Zhao Y., Corti C., Ficetola G.F., Manenti R.Lunghi E., Giachello S., Zhao Y., Corti C., Ficetola G.F., Manenti R.	Photographic database of the European cave salamanders, genus Hydromantes	Scientific Data, 7: 171		
2020	Lunghi E., Giachello S., Manenti R., Zhao Y., Corti C., Ficetola G.F., Bradley J.G.	The post hoc measurement as a safe and reliable method to age and size plethodontid salamanders	Ecology and Evolution, 10: 11111-11116		
2020	Lunghi E., Manenti R., Cianferoni F., Ceccolini F., Veith M., Corti C., Ficetola G.F., Mancinelli G.	Interspecific and interpopulation variation in individual diet specialization: Do environmental factors have a role?	Ecology, 101: e03088	Foraging tactics	observational
2020	Lunghi E., Cianferoni F., Ceccolini F., Zhao Y., Manenti R., Corti C., Ficetola G.F., Mancinelli G.	Same diet, different strategies: Variability of individual feeding habits across three populations of ambrosi's cave salamander (Hydromantes ambrosii)	Diversity, 12: 180	Foraging tactics	observational
2020	Ficetola G.F., Lunghi E., Manenti R.	Microhabitat analyses support relationships between niche breadth and range size when spatial autocorrelation is strong	Ecography, 43: 724-734		
2020	Salvidio S., Costa A., Oneto F., Pastorino M.V	Variability of a subterranean prey-predator community in space and time	Diversity, 12: 17	Foraging tactics	observational

2020	Bruni G.	Tail-straddling Walk and Spermatophore Transfer in Hydromantes italicus: First Observations for the Genus and Insights about Courtship Behavior in Plethodontid salamanders	Herpetological Review, 2020: 673–680	Intraspecific social behavior, courtship, mating and parental cares	observational
2020	Lunghi E., Corti C., Mulargia M., Zhao Y., Manenti R., Ficetola G.F., Veith M.	Cave morphology, microclimate and abundance of ive cave predators from the Monte Albo (Sardinia, Italy)	Biodiversity Data Journal, : e48623		
2020	Brigsøe H., Brigsøe A.	Erste Beobachtung einer Paarung beim Supramonte-Höhlensalamander, Speleomantes supramontis in seinem natürlichen Lebensraum	Amphibia, 19: 7-15	Intraspecific social behavior, courtship, mating and parental cares	observational
2021	Lunghi E., Cianferoni F., Giachello S., Zhao Y., Manenti R., Corti C., Ficetola G.F.	Updating salamander datasets with phenotypic and stomach content information for two mainland Speleomantes	Scientific Data, 8: 150		
2021	Lunghi E., Bacci F., Zhao Y.	How can we record reliable information on animal colouration in the wild?	Diversity, 13: 356		
2021	Lunghi E., Cianferoni F., Merilli S., Zhao Y., Manenti R., Ficetola G.F., Corti C.	Ecological observations on hybrid populations of european plethodontid salamanders, genus speleomantes	Diversity, 13: 285	Foraging tactics	observational
2021	Salvidio S., Costa A., Oneto F.	Size-related boldness is not altered by stomach flushing in a terrestrial salamander	Amphibia Reptilia, 42: 377-382	Cognitive ecology and personality	experimental
2021	Rosa G., Costa A., Renet J., Romano A., Roner L., Salvidio S.	Energy storage in salamanders' tails: the role of sex and ecology.	Science of Nature 108:27.		
2021	Schulz V., Gerhardt P., Stützer D., Seidel U., Vences M.Schulz V., Gerhardt P., Stützer D., Seidel U., Vences M.	Lungless salamanders of the genus speleomantes in the solling, germany: Genetic identification, bd/bsal-screening, and introduction hypothesis	Herpetology Notes, 14: 421-429		
2021	Crochet P.-A.Crochet P.-A.	Nomenclature of European plethodontid salamanders: Speleomantes Dubois, 1984 has precedence over Atylodes Gistel, 1868	Amphibia-Reptilia, 28: 170-172		

2021	Lunghi E., Sisino L., Mulargia M., Manenti R., Ficetola G.F.	Censimento dei siti ipogei frequentati dai pletodontidi europei: cinque anni di segnalazioni	Atti XII Congresso Nazionale Societas Herpetologica Italica. Rende 1-5 ottobre 2018. Pp. 222-225. Tripepi S. (Ed.)		
2021	Lunghi E., Corti C.	Predation of European cave salamanders (Hydromantes) by the spider Meta bourneti	Spixiana, 44: 54	Interactions with predators	observational
2022	Delunas C.	From wax to polymer clay in an amphibian model	Museologia Scientifica, 16:78-80.		
2022	Lunghi E., Cianferroni F., Corti C., Zhao Y., Manenti R., Ficetola G.F., Mancinelli G.	The trophic niche of subterranean populations of Speleomantes italicus	Scientific Reports, 12(1), 18257.	Foraging tactics	observational
2022	Rosa G.	Optimizing monitoring of an endemic terrestrial salamander (Speleomantes ambrosii): comparing cost-effectiveness of different methods for abundance estimation	Rendiconti Lincei - Scienze Fisiche e Naturali, 33: 807-813.		
2022	Lunghi E., Corti C., Biaggini M., Merilli, S. Manenti R., Zhao Y.H., Ficetola G.F., Cianferoni F.	Capture–mark–recapture data on the strictly protected Speleomantes italicus	Ecology, 103. DOI10.1002/ecy.3641		
2022	Lunghi E., Cianferoni F., Corti C., Zhao Y.H., Manenti R., Ficetola G.F., Mancinelli G.	The Trophic Niche of Two Sympatric Species of Salamanders (Plethodontidae and Salamandridae) from Italy	Scientific Reports, 12. DOI10.1038/s41598-022-21819-8	Foraging tactics	observational
2022	Rosa G., Bosio M., Salvidio S., Costa A.	Foraging success is differently affected by local climate in two syntopic forest-dwelling salamanders.	Ethology Ecology Evolution, 35:424–433. doi.org/ 10.1080/03949370.2022.2094470.	Foraging tactics	observational
2022	Rosa G., Salvidio S., Costa A.	European Plethodontid salamanders on the forest floor: Testing for age-class segregation and habitat selection.	Journal of Herpetology, 56: 27-33. DOI: 10.1670/20-151	Intraspecific social behavior, courtship, mating and	observational
2022	Costa A., Rosa G., Romano A., Salvidio S.	Weighted individual-resource networks in prey-predator systems: the role of prey availability on the emergence of modular structures.	Integrative Zoology, 17: 115-127. doi.org/10.1111/1749-4877.12520	Foraging tactics	observational
2022	Lunghi E., Manenti R., Cimmaruta R.	The identity of an allochthonous Pyrenean population of Speleomantes cave salamanders	Salamandra, 58: 67-70.		

2022	Lunghi E.	Doubling the lifespan of European plethodontid salamanders.	Ecology, 103(2), e03581.
